# Antibiotic prescription, dispensing and use in humans and livestock in East Africa: does morality have a role to play?

**DOI:** 10.1007/s40592-024-00208-z

**Published:** 2024-10-17

**Authors:** Edna Mutua, A. Davis, E. Laurie, T. Lembo, M. Melubo, K. Mnzava, E. Msoka, F. Nasua, T. Ndibohoye, R. Zadoks, B. Mmbaga, S. Mshana

**Affiliations:** 1 School of Social and Political Sciences, School of Health and Wellbeing, University of Glasgow, 11 Chapel Lane, Glasgow, G11 6EW UK; 2 School of Geographical and Earth Sciences, University of Glasgow, Glasgow, UK; 3 The Boyd Orr Centre for Population and Ecosystem Health, Institute of Bioaffiliationersity, Animal Health & Comparative Medicine, College of Medical, Veterinary and Life Sciences, University of Glasgow, Glasgow, UK; 4 Kilimanjaro Christian Medical Centre/Kilimanjaro, Kilimanjaro Clinical Research Institute, Christian Medical University College, Moshi, Tanzania; 5 Department of Microbiology and Immunology, Catholic University of Health and Allied Sciences/Bugando Medical Centre, Mwanza, Tanzania; 6 University of Sydney, Sydney, Australia

**Keywords:** Antimicrobial resistance, Morality, Health systems, One health, East Africa

## Abstract

**Background:**

Antimicrobial resistance (AMR) is a global threat to human and livestock health. Although AMR is driven by use of antimicrobials, it is often attributed to “misuse” and “overuse”, particularly for antibiotics. To curb resistance, there has been a global call to embrace new forms of moral personhood that practice “proper” use, including prescription, dispensing and consumption of antimicrobials, *especially* antibiotics. This paper seeks to reflect on complex questions about how morality has become embedded /embodied in the AMR discourse as presented in the data collected on antimicrobial prescription, dispensing and use in human and livestock health in Tanzania, primarily focusing on antibiotics.

**Methods:**

This reflection is anchored on Jarrett Zigon’s morality framework that is comprised of three dimensions of discourse; the institutional, public, and embodied dispositions. The data we use within this framework are derived from a qualitative study targeting human and animal health care service providers and community members in northern Tanzania. Data were collected through 28 in-depth interviews and ten focus group discussions and analysed through content analysis after translation and transcription. In addition, a review of the Tanzania’s National Action Plans on antimicrobial resistance was conducted.

**Results:**

Application of the framework demonstrates points of convergence and divergence in the institutional morality discourse articulated by the Tanzania National Action Plans, the public discourse and the embodied dispositions/ lived experiences of human and animal health care service providers and community members. We demonstrate that AMR is not just associated with “inappropriate” behaviour on the part of drug prescribers, dispensers, and users but also with shortcomings in health systems and service delivery.

**Conclusion:**

Antibiotic dispensing and use practices that may be associated with the development of AMR should not be viewed in isolation from the broader health context within which they occur.

**Supplementary Information:**

The online version contains supplementary material available at 10.1007/s40592-024-00208-z.

## Introduction

Antimicrobial resistance (AMR), which cumulatively refers to pathogen resistance to antibiotics, antiprotozoals, antifungals and antivirals, is a global human and animal health concern (WHO 2016). Of the four types of antimicrobials, more emphasis on curbing resistance has been placed on antibiotic resistance (ABR) which has both political and biomedical connotations (WHO 2016). ABR is expected to cause ten million deaths and economic losses of $100 trillion (O’Neill 2014; Founou et al. 2017) with Asia and Africa, in particular, projected to suffer 4.7 million and 4.1 million deaths respectively by 2050, (O’Neill 2014). A recent study further shows that in 2019, 4.95 million deaths were related to AMR while 1.27 million were directly linked to antibiotic resistance; majority of which occurred in sub-Saharan Africa and south Asia (Murray et al. 2022).

Antimicrobial use ranges from disease prevention and treatment in both humans and animals (WHO 2016) to growth promotion in food animals (Van Boeckel et al. 2015; WHO 2016). Such widespread antimicrobial use can lead to the development of resistance in bacteria in humans and livestock. Exposure to resistant bacteria and genes or antimicrobial residues can occur through foods or their derivatives (both human foods and animal feed); contact with humans, human fluids and waste, animals, animal fluids and manure; and the broader environment (e.g. contaminated soil and water) (Van Boeckel et al. 2015; Founou et al. 2016; Graham et al. 2017). The interconnectedness of human and animal processes in the development and spread of resistance has led to AMR being considered a One Health issue requiring combined intervention from human health, livestock health and environmental dimensions (WHO 2016; Chandler 2019). However, despite global concerns regarding excessive use of antimicrobials, access is highly variable. For example, in LMIC countries, an estimated 5.7 million preventable human deaths occur due lack of access to antimicrobials (Daulaire et al. 2015; Nonga and Katakweba 2015).

Despite issues of access, resistance is commonly framed by the global health community as driven by “misuse” and “overuse” of antimicrobials, often through individual use, across countries (O’Neill 2014; WHO 2016). AMR discourse has predominantly been championed by developed countries who often have a louder voice and more resources in global discussion and policy (Chandler et al. 2016). Current international discourse and policy to adopt “optimal”/ “rational”/ “proper”/ “prudent” use of antimicrobials within antimicrobial stewardship programmes has led to the emergence of critical theoretical and material questions about how AMR is produced, transmitted, and sustained (Caudell et al., 2018). Likewise, it calls into question, what constitutes “optimal”/ “rational”/ “proper”/ “prudent” prescription, dispensing and use of antimicrobials, particularly antibiotics, in practice. The use of these terms presupposes that the opposite is possible; those medicines can be used “sub-optimally”/ “irrationally”/ “improperly” or “imprudently”. This advances ideals on “right” versus “wrong” ways of drug use thus introducing a moral discourse on drug use as well as a moral position on antimicrobial stewardship. This begs the question about how the global health community develops new forms of moral personhood on how medicines should be prescribed, dispensed and utilized (Chandler et al. 2016).

Drawing from Jarrett Zigon’s (2010) framework on morality, in which he suggests there are three interrelated aspects of morality, including the institutional sphere, public discourse, and the embodied dispositions or moral habitus of the everyday, this paper seeks to reflect on complex questions about how morality has become embedded and embodied in AMR discourse. We use qualitative data collected from northern Tanzania on AMR and specifically antibiotics to explore this topic in-depth.

## Background

### The rise of global discourse on AMR

Biomedically, antibiotics are the most developed and widely used of the four antimicrobials (Gyssens 2018). Despite recent developments in the past few years (see Andrei et al. 2019 https://www.ncbi.nlm.nih.gov/pmc/articles/PMC7086080/), compared to early advancements, development of new compounds has been relatively slow since the 1990s (WHO 2016). At their inception, antibiotics, particularly penicillin, were considered “miracle medicines” and “magic bullets” capable of eliminating infectious diseases (Chandler et al. 2016; Kazanjian 2017). This perception led to unrestrained prescription and increased use as more varieties were introduced and became easier to produce and access in the Global North (Kazanjian 2017).

Alexander Fleming recognized very early that widespread use of antibiotics could lead to the development of resistance in bacteria and efforts to regulate antibiotic prescription in the human health sector began as early as the 1950s (Kazanjian 2017). Through to the 1970s, individual hospitals and/or national institutions such as the United States Centres for Disease Control (CDC) pushed for adoption of regulatory measures on a broader scale (ibid.). In the late 1970s and 1980s, there were various intergovernmental discussions facilitated by the United Nations about drug policy (Streky 1985). In 1985, a World Health Organization (WHO) conference on “rational use of drugs” aimed discussion at malpractices in the pharmaceutical sector as well as misuse through prescription and patient use (WHO 1987). From 1986 to 2014, the World Health Assembly passed three further resolutions on “rational use of drugs” and two resolutions on curbing AMR with the latter setting the background for the development of the WHO’s 2015 Global Antimicrobial Resistance Action Plan (GAP) (WHO 2014). The plan proposes a five-pronged strategy for combating AMR combining: (1) awareness creation; (2) surveillance and evidence generation; (3) infection prevention and control (IPC) in communities and health facilities; (4) optimal use of antimicrobials; and (5) sustainable financing to facilitate development of new antimicrobials, vaccines and diagnostics (WHO 2016). WHO member states are encouraged to develop and implement a National Action Plan (NAP) on AMR control. While ostensibly nationally led, the expectation is that any NAP is based on the five objectives identified by WHO. Here then, geopolitically, national autonomy for tackling AMR is largely subsumed by the WHO and the voices of developed countries operating in a global landscape critiqued for protecting the wealthy from the biosecurity risks associated with the boundaryless nature of AMR (Chandler 2019). Tanzania, as a member state, developed their first NAP in 2017, which, despite capturing the country’s specific challenges and recommending practical actions to guide its own national level efforts of combating AMR (MoHCDGEC 2017), has been built around the five objectives identified by the WHO GAP. In the Tanzanian context, pharmacists and drug dispensers (at accredited drug dispensing outlets (ADDO) as well as agro-vet shops legally dispense antimicrobials with prescriptions (or recommendation from a veterinarian in the case of livestock). However, sale of antibiotics commonly happens outside these confines, particularly at ADDOs without prescription or agro-vets without veterinary advice (see Virhia et al. 2023 for detailed description of the health context in relation to antimicrobials in Tanzania).

Globally, then, conversations, campaigns, plans, and policies concerning AMR control are rooted in a discourse of “optimal”/ “rational”/ “proper”/ “prudent” use, where the language of protection and conscientiousness dominates and, central to that, the individualisation of action has emerged as the commanding approach (Broom et al. 2020). However, this language and approach needs to be called into question: does protecting “vulnerable medicines” supersede protecting “vulnerable people” who have limited access to antimicrobials, and antibiotics in particular (Chandler et al. 2016)? Global efforts to curtail AMR aim to protect certain antibiotics that are defined ‘vulnerable’. These compounds are for the most highly resistant infections for which other antibiotics fail (ibid.). Yet, the vulnerability of drugs will vary geographically dependent on the diseases of concern in any given location. This ‘global’ approach raises the question that if vulnerable medicines should be saved, whom are they to be saved for? And, central to that, how are the behaviours of some becoming judged in the moralising discourse inherent in “proper” use? That is to say, if medicines are to be used “rationally” / “properly”/ and “prudently” then the opposite is therefore possible, and medicine use can be “sub-optimal”/ “irrational”/ “improper” or “imprudent”. The “right” or “wrong” of drug use and hence antimicrobial stewardship has therefore become a dialogue about morality.

## Morality of AMR framed through anthropological debates

Morality within anthropology often relates to the research ethics governing anthropologists’ motives and actions or it can be called into play to assess or discuss the lives and experiences of research subjects (Butt 2002; Fassin 2008). As an anthropological study area, morality has recently been revived as an active topic of debate (Stoczkowski 2008; Biehl and Moran-Thomas 2009; Beldo 2014; Cassaniti and Hickman 2014; Shweder and Menon 2014; Wong 2014; Zigon and Throop 2014) and has been extensively addressed by Zigon (2010) in response to Fassin and Stoczkowski’s public debate (see special issue of *Anthropological Theory* 2008). The origins of these debates can be traced back to Durkheim’s influential conceptualization of society as the principal moral authority (Eberhardt 2014). This formulation has been criticized for conflating “the social” with “the good” in ways that did not allow for distinct conceptualization and study of ethics thereby stagnating the subject area (Laidlaw 2002). Other anthropological discourses view morality from “relativism”, “universalism” or “contextualism” perspectives (Shweder 2004, 2012; Zigon 2010; Beldo 2014). Some of the challenges associated with anthropologists’ interest in morality include a lack of agreement on what constitutes morality; how morality and ethics are linked; how to identify and ground morality issues in ethnographies; and how to talk about the topic (Cassaniti and Hickman 2014). However, these challenges and debates have furthered anthropological engagements into the subject area leading to important epistemological (and methodological) contributions. While it is outside the scope of this paper to fully review the current lively and productive revival of morality as a topic within our discipline, it is important to note how our study of AMR has been shaped by these debates.

Anthropological perspectives on morality can reveal complex moral issues that various societies or communities and their members face, be it those found within (i.e. that societies contend with/create locally) or which members find themselves confronting as a result of global/outside forces (Butt 2002; Turner 2003; Zigon 2010; Cassaniti 2014; Hickman 2014). Zigon’s methodological framework of social morality is influential here as he suggests there are three interrelated aspects of morality including the *institutional sphere*, *public discourse* and the *embodied dispositions* or moral habitus of the everyday (Zigon 2010). The framework not only provides theoretical insights, but also facilitates *application* of the theory to real-time, moral matters found within given community contexts. For example, Zigon builds and applies his approach to ethnographic work on Russian drug rehabilitation facilities (ibid.).

Institutions, whether formal or informal, have their own moral claims and degrees of power to enforce them on individuals. For example, institutions such as national governments and ministries of health can have a moral standpoint in the context of AMR when it’s a consequence of antibiotic misuse by health sector actors. Within the public discourse of morality are found the moral claims, conceptions, and beliefs of society, which may or may not support the same moralities as a society’s institutions (e.g. health and education centres). While constantly in dialogue with one another, Zigon suggests that this is where challenges to society, the state and its institutions are found (Zigon 2010). Thus, we support the notion that morality within public discourse can be espoused by those who may support as well as challenge broader social morality, including through media, community leaders or forums, the arts, in stories or even through academics, like anthropologists.

Zigon’s third aspect of morality is found in our embodied dispositions, i.e. how individuals live their lives in socially acceptable ways within their geographic space. Embodied disposition may/may not be a conscious reflection or enactment which Zigon compares to Mauss’ original version of habitus (Mauss 1979; Farnell 2000), but in slight contrast to the ubiquitous social concept greatly popularised by Bourdieu (Zigon 2011). Compared to Bordieu’s habitus, which in simplified terms has come to mean a group of people’s social habits, practices, dispositions and tastes, Mauss’ habitus is seen in terms of the disposition of the body relative to a particular society, a disposition that is dynamic and performed, but consciously so, and is therefore tied to moral and ethical choices. Bourdieuian habitus is less conscious, and more driven by commonality of custom but not necessarily related to moral choices (see Farnell’s (2000) discussion of Mauss’ 1935 work; and Mahmood’s (2011) critique). For Zigon, habitus is tied to ethics. As Das suggests (2006), it is in everyday life and language that we can understand moral experience and ethical practice (Zigon and Throop 2014).

Zigon’s framework also introduces the concept of a “moral breakdown”, understood as a moment when individuals shift from a non-reflective application of embodied morality to a conscious one (Zigon 2007, 2010). This occurs when someone is faced with an “ethical moment” presenting itself as a moral dilemma (Zigon 2007, 2010). In these moments, individuals consciously decide on how to act in a manner they consider good or right depending on context, while simultaneously exposing motivations behind their decisions/actions (Robbins 2007; Zigon 2010). As moral breakdowns are likely to occur from time to time, resulting in different choices and actions, morality is thus not static but a lifelong exercise of “adjusting and readjusting” (Zigon 2010: 9).

Zigon presents his work in terms of a drug rehabilitation program in Russia, a non-western context, whereas our study on AMR from Tanzanian communities and its tiered health system concerns an even more amorphous, complex space, testing the robustness of the framework. We are applying Zigon’s framework in an African setting, where health is often experienced from a pluralistic, socio-culturally mediated sphere (Sunday Idemudia and Adedeji 2023; Scheper-Hughes and Lock 1987; Rekdal 1999; Schoenbrun 2006) yet where the health system is one based on a western, bio-medical framework (Virhia et al. 2023; Higgins 2016) and influenced by global health institutions. Thus, we find Zigon’s framework useful and applicable for numerous reasons presented above. We find exploring morality from overlapping and intertwined scales, institutions, and narratives as Zigon explicates, particularly compelling. In our application, we therefore consider the positions articulated by the WHO’s GAP and Tanzania’s NAPs on antimicrobial resistance as the key discourse related to the institutional dimension of Zigon’s framework. Zigon’s public discourse is paralleled by AMR discourses articulated by human and animal health professionals and local communities across northern Tanzania. Finally, embodied dispositions are drawn from the views articulated by individual persons on drug prescription, dispensing and use in their day-to-day lives and with direct implications on bodies and health. Zigon’s framework clearly reflects the myriad views and culturally specific experiences and plurality of body, health, wellbeing, and healthcare in this setting.

## Materials and methods

This research presents findings from a study conducted in northern Tanzania titled “Supporting the National Action Plan for Antimicrobial Resistance in Tanzania (SNAP-AMR)”. We present a summary of Tanzania’s 2017–2022 and 2023–2028 AMR NAPs achieved through a review and discourse analysis of the two documents. We also present qualitative data from this study conducted across multiple tiers of the Tanzanian health system incorporating both community-based and hospital settings. Social science data collection for SNAP-AMR was conducted in multiple phases with phase 1 or “preliminary” data collected from April-May 2019 within communities and April 2019-February 2020 in the higher tiers of the health system and a second phase of data collection occurring between January-July 2021. Additional data collection was conducted through a mixed-methods PhD project (see Loosli 2024). Details on our methods and tools can also be found via Lembo et al. (2024). Detailed methods and initial findings related to issues of heath equity and healthcare access (Davis et al. 2022; Virhia et al. 2023), about stated preference data (Nthambi et al. 2023), and on the project as a whole (Virhia et al. 2024; Loosli 2024) have been published. Tanzania was considered a suitable study site evidenced by their political will to control AMR and through conducting a situation analysis on AMR (Nonga and Katakweba 2015), development of a national action plan for the containment of AMR (MoHCDGEC 2017) and their recognized need to strengthen capacity for AMR research all which allowed for implementation of this collaborative project. The preliminary data collection, from which this paper draws, took place at community levels in Mwanga, Ngorongoro, and Misungwi districts in Kilimanjaro, Arusha, and Mwanza regions respectively. Each region represented a specific agricultural production system with smallholder farmers (Kilimanjaro), pastoralists (Arusha) and agropastoralists (Mwanza). Three purposefully sampled villages (one from each district) met the following criteria for inclusion: having key stakeholders within the human and veterinary health sectors (e.g. nurses/ clinical officers from local health facilities, veterinary/livestock field officers, community (human and animal) health workers) and facilities for health care (either health centre, dispensary, clinic, and/or agrovet shop or pharmacy). Interviews were conducted by a field team of Tanzanian social science trained research assistants and a social scientist team lead all under the direction of the main authors. The team was composed of 5 Tanzanian field based social science assistants and lead working in the health sector with some members of the team drawn from the study sites. The lead authors include a Kenyan anthropologist serving as a postdoc at the time of the study and an American anthropologist with 25 years experience working in the region.

A total of 10 Focus Group Discussions (FGDs) with 84 discussants and two in-depth interviews (IDIs) were conducted at the community level (Table 1). Three FGDs were conducted with community members about treatment of common human diseases, drug access, and utilization. A further three discussed these topics from animal health perspectives. Three more FGDs were conducted with human health care providers (clinical officers, nurses, pharmacy attendants, community health workers) from the three districts and one was conducted with animal health care providers (the livestock field officers and attendants in agrovets). We did not conduct IDI’s with pharmacist because the sites we visited in this phase did not have them. Due to how few animal health respondents were present in two communities, two IDIs were held instead. All FGDs and IDIs were conducted in Swahili except in Ngorongoro where the Maa language was used.

**Table 1:** Study participants

	Data collection method	Types of participants	Number of FGDs/IDIs	Number of participants	District
1	Focus group discussions (FGDs)	Community members (human health)	1	6	Mwanga
			1	7	Misungwi
			1	12	Ngorongoro
		Community members (animal health)	1	10	Mwanga
			1	8	Misungwi
			1	11	Ngorongoro
		Human health care providers	1	5	Mwanga
			1	11	Misungwi
			1	8	Ngorongoro
		Animal health care providers	1	6	Ngorongoro
2	In-depth interviews (IDIs)	Animal health care providers	1	1	Mwanga
			1	1	Misungwi
		Human health care providers	26	26	Kilimanjaro and Misungwi

Table 1 details the study participants who discussed treatment of common human and animal conditions, including access to and use of medicines.

Representing the higher tier of the health system, hospital interviews included 16 medical doctors from a regional referral hospital, and 10 from district hospital (Table 1). Doctors were drawn from medical, paediatric, orthopaedic, and ophthalmology departments. They were interviewed on their awareness of AMR drivers, their prescribing practices, and patient access and use of medicines. The interviews were conducted in English or Swahili (based on interviewee preference).

All discussions and interviews were audio recorded with complementary field notes taken. All audio files in Swahili or Maa were translated into English and transcribed for analysis. The community and hospital level data sets were separately managed and analysed into emergent themes on NVivo 12 through deductive and inductive data analysis (Creswell and Creswell 2017). Inductive analysis focuses on developing themes based on the patterns coming from the collected data themselves, while deductive analysis focuses on analysis guided by data collection tools and literature (ibid.).

## Results

Our results are organized into three main sub-sections, mirroring the three components of morality discourses advanced by Zigon (2010). The first section covers institutional discourse on AMR as presented in the Tanzania’s NAP. The second section explores the public discourses of AMR by human and animal health care providers and community members. The final section addresses the embodied dispositions of animal and human health care service providers and community members when forced to “step out of their everyday mode of moral being and into the ethical moment” (Zigon 2007: 144).

### The institutional policy discourse

The 2017–2022 Tanzania NAP begins by stating, *“The plan is coherent to the WHO Global Action Plan on antimicrobial resistance ….”* (2017, Page viii). The NAP adopts WHO’s five strategic objectives, one of which addresses the question of “optimal” use of antimicrobials. The challenge of AMR is first framed in the NAP as one of undesirable behaviour, manifesting in the form of inappropriate use of antimicrobials in human, animal, and plant health. Proposals are made on how to curb AMR through forms of institutional, public, and individual behaviour control related to drug manufacture, distribution, and use as demonstrated below:

*“Interventions to* ***optimize*** *the use of antimicrobial agents in human and animal health will include* ***strengthening regulatory authority*** *for controlling quality*,* distribution and use of antimicrobial agents; strengthening patient and health care provider* ***compliance***; *reducing the prevalence of* ***substandard*** *medicines for both human and veterinary use and* ***inappropriate*** *or* ***unregulated*** *use of antimicrobial agents in agriculture; and strengthening stewardship for antimicrobial use in health facilities* ***by providing evidence based prescribing and dispensing*** *of standards of care. Stewardship will include* ***monitoring and evaluating*** *the use and consumption of antibiotics at all levels.” Page ix*.

The NAP utilizes terms associated with behaviour control through enforcement in institutions, the public, and individuals including, “regulatory authority”, “regulation”, “compliance”, “substandard”, “restrictions” and “legal instruments (Acts, Regulations and Guidelines)”. This language demonstrates the power hierarchies and relations that exist between government agencies and other institutions, the public, and/or individuals. It is these varying degrees of power that can be used to construct and enforce a given moral standpoint related to drug manufacture, distribution, and use.

The AMR challenge is also framed as one of lack of knowledge as shown below:

*“In Tanzania*, ***public awareness of antimicrobial resistance is still a challenge*** *as currently these programmes are not in place.* ***Patients’ knowledge*** *of prescribed antimicrobials*,* including routes*,* dosages*,* frequency of administration*,* duration of use*,* and the importance of adhering to treatment even if they feel better*, ***is limited***.*” Page 5*.

Thus, the NAP calls for raising AMR awareness and the need to promote behaviour change in a bid to enhance practice of “appropriate” behaviours. It aptly states that, *“This National Action Plan addresses actions needed to be taken in order to combat antimicrobial resistance (AMR) in the country. It is* ***obligatory*** *to* ***raise awareness*** *of antimicrobial resistance and* ***promote behavioral change*** *through public communication programmes that targets human*,* animal and plant health.” (Page vi).* In so doing, the NAP emphasises the interrelatedness between human, animal, and plant health hence the importance of adopting a One Health approach in curbing AMR.

The 2023–2028 NAP in similar vein states that, *“The National Action Plan on Antimicrobial Resistance (NAP-AMR*,* 2023–2028) envisage to continue with implementation of the five strategic objectives in line with the Global Action Plan on AMR*,*” (Page x*, GoT 2023. Further, “*The WHO Implementation Handbook for National Action Plan on Antimicrobial Resistance 2022*,* provided technical guidance and stepwise approach for the development of this document.” (Page ix*, GoT 2023*).* Both statements demonstrate the level of influence that global AMR policy discourse has at national level.

### The public discourse

Below we present AMR discourses as expressed through conversations with health facility workers across tiers, livestock healthcare workers, and community members. Of the 16 medical doctors interviewed in the referral health facility, only two were familiar with the NAP. While findings presented here may not be directly influenced by the NAP, they reverberate the institutional discourse.

Health providers from the referral hospital framed AMR challenges as influenced by the behaviours of medical practitioners, pharmacists, and community members (i.e. patients), through the actions they take in drug prescription, dispensing, and use respectively. While recognising the broad influences, they principally saw AMR as driven by community misuse brought about not by lack of resources but a lack of education:

*“Most of the patients in this place are layman*,* the majority don’t know even that there are medicines called antibiotics.” Human health care provider*,* Misungwi district IDI 23.*

Doctors felt that even if they explained, it would have little impact, as noted:

*“Yes*,* we do communicate but patients don’t understand*,* and they don’t take it seriously even if you talk to them about it.” Human health care provider*,* Misungwi district IDI 18.*

*“Some understand*,* but majority you can talk to them… you can tell a patient “I won’t prescribe for you any medicine because this is just a normal cough*,* it will pass”*,* but s/he goes around … [will] go and take amoxicillin*,* you just wonder you didn’t prescribe her/him but s/he takes the medicines.” Human health care provider*,* Misungwi district IDI 21.*

Health service providers working at community level, similarly framed AMR as associated with self-treatment:

*“Self-treatment should stop because a person does not know what he is suffering from.” Human health care provider*,* Mwanga district*,* FGD 01.*

Here, health care providers framed self-treatment as negative due to the potential for misdiagnosis and mismanagement of disease which they believed endangers lives and compromises the desired health outcomes of people.

Similarly, self-treating one’s animals was framed as inappropriate by veterinary officers because of risks of misdiagnosis, overdosing, or underdosing which can cause negative health outcomes for livestock. Further, it was considered contravention of the country’s veterinary regulations:

*“You know the law restricts any unauthorized person from treating animals*,* it requires the Livestock Field Officer to do so. But*,* because these people have the experience in treating animals for a long time*,* that is why they treat them themselves.” Animal health care provider*,* Ngorongoro district*,* FGD 05.*

In agreement with observations made by health service providers, community members acknowledged that self-treatment (humans and livestock) was a common practice. They framed it as a means of obtaining care when the health system does not meet their needs and as an expression of choice and agency based on their circumstances. Farmers from all districts framed treating their own livestock not as a contributor to AMR but as a solution to the lack of reliable veterinary services, as a means of circumventing high costs of professional veterinarians, and as an opportunity to utilize their own knowledge to enhance their livestock’s health.

### The embodied dispositions

The embodied dispositions of human and livestock health workers and community members were expressed through their reported daily life experiences. Among medical doctors from the referral health facility, there was agreement that there are actions taken in the line of duty that could be linked to ABR. Prescribing antibiotics for conditions for which they are not required, for example, common colds or as precautionary prophylactics, was cited:

*“Doctors tend to be cautious… for example let’s say a child has a throat infection. I think it’s viral and I should send the mom home with panadol and [advise them] to just get some rest or fluids*,* but if I am wrong and it is bacterial this child will get worse. So as the doctor I will be like why should I risk? You know what? Just take this antibiotic because it is 50–50.” Medical doctor*,* Referral facility*,* IDI 13.*

Use of clinical diagnosis without conducting any additional tests due to lack of laboratories or the narrow range of available tests was linked to antibiotic over-prescription. Costs associated with diagnostic testing added further constraints to patients and an additional delay in the care seeking process:

*“Take an example of malaria*,* the use of anti-malarials I think has gone down remarkably because of improved diagnostics. After the rapid malaria test [was introduced]*,* anti-malarial use has gone down very much. Initially everybody used to know that if you have fever it’s probably malaria but we all know now that it’s not. So*,* if it’s not malaria it’s an infection and if it’s an infection*,* infection is treated with antibiotics*,* it doesn’t matter if it’s viral. I think in a way having malaria rapid tests have reduced the use of antimalarials but as well increased the use of antibiotics.” Medical doctor*,* Referral facility*,* IDI 04.*

Accessibility and affordability of diagnostics by both health facilities/providers and patients/farmers are critical to treatment decisions. However, in LMICs, testing capacity can be severely constrained (Horton et al. 2018; Sayed et al. 2018). Reasons are multiple, ranging from understaffing of qualified laboratory staff and varying states of laboratory infrastructure to inconsistent supply of laboratory consumables (*ibid.;* Virhia et al. 2023).

Even with a diagnosis, issues of underdosing or over-prescribing, for example of a single or multiple antibiotic course, can contribute to the development of resistance. In a system where patients are required to seek care in dispensaries and health centres first, before onward referral to district and regional health facilities, there is a risk of over-exposure to antimicrobials as they move from one health facility to another in search of care:

*“The source [of resistance] could be that because we are a referral hospital*,* so most patients that we receive have already passed by other health facilities and they have already used many drugs.” Medical doctor*,* Referral facility*,* IDI 12.*

“Normalizing” antibiotic prescription without commensurate consciousness of the risk of resistance was also identified by doctors as a potential cause of AMR:

*“It is true I can prescribe antibiotics when not needed because there are a lot of patients and I need to leave. At that time.…. I might prescribe an antibiotic that most people are usually prescribed for.” Medical doctor*,* Referral facility*,* IDI 03.*

The latent risk in clinicians “normalizing” antibiotic prescription was that it creates an expectation in patients that they ought to receive medicines, particularly antibiotics, every time they visit a health facility.

#### Referral health facility workers’ views on pharmacists

Medical doctors further identified pharmacists as actors whose actions can be drivers of AMR. Pharmacists often play a doctor’s role of diagnosing and prescribing medicines, including antibiotics, to persons who come to their outlets. They arrive at a diagnosis by relying on explanations provided to them by the buyers, as one doctor detailed.

*“There is another practice which I normally witness. Someone can come to the pharmacy and then they start explaining their problem to the pharmacist*,* that I have one*,* two*,* three*,* four. The pharmacist listens to them and then at the end says I will give you this [medicine]. That is something which is not ethical*,* which is not right.” Medical doctor*,* Referral facility*,* IDI 02.*

Pharmacists also sell antibiotics to buyers with and without prescriptions. Quantities sold depend on buyer requests or affordability of medicines, thus sale of partial doses is possible (Minzi and Manyilizu, 2013). Doctors also emphasised that pharmacies are motivated by profit and are reliant on making sales:

*“…a person comes and they want to buy antibiotics let’s say which cost five thousand Tanzanian shillings (2.2 USD)*,* six thousand (2.6 USD) … or 25 thousand (10.8 USD). That person does not have any prescription. The pharmacist is a businessperson. Will they seriously refuse the 30 thousand (12.9 USD) because the patient does not have a prescription?” Medical doctor*,* Referral facility*,* IDI 02.*

Pharmacists may also trade in expired or substandard/counterfeit medicines (un)intentionally as one doctor stated:

*“Nowadays there are fake antibiotics…. It is very difficult to be able to discover that this a fake and this the original medicine.” Medical doctor*,* Referral facility*,* IDI 03.*

Alongside concerns about counterfeits, doctors also raised the issue of pharmacists trading in expired medicines. Part of the strategies utilized by pharmacists to make the medicines appear usable to buyers was changing containers/boxes to one with viable dates.

#### Human health care workers views on community members

Community members were the final category of actors identified as making decisions that drive antibiotic resistance. Doctors described use of antibiotics for conditions for which they are not required, such as the common cold, or in ways that they should not be used.

“*There are some patients I have come across*,* let’s say you have prescribed for him maybe flucamox*,* an antibiotic*,* you give it to him to take [orally] and he invents another way. He takes the capsule and removes the powder inside then puts it on the wound instead of taking [orally]. I think one [cause of resistance] is changing medicine usage from how it’s supposed to be taken and using it the way he wants.” Medical doctor*,* Referral facility*,* IDI01.*

Patients also put pressure on doctors to prescribe certain types of medicines based on prior experience even though they may not be appropriate for their current condition. This notion is also driven by patients’ perception that a positive health outcome is reliant on the use of medicines:

*“Patients might come with a disease which you think does not require antibiotics but for them to feel better*,* they feel that they need to be prescribed antibiotics. So*,* they might pressure you. The satisfaction of the patient might depend on you prescribing antibiotics.” Medical doctor*,* Referral facility*,* IDI 02.*

Health service providers across tiers indicated AMR could be associated with patient self-diagnosis and treatment after they fail to take the full antibiotic course prescribed because they start to feel better:

*“I sometimes spend some time at the pharmacy up there. I have never seen a patient come with a prescription form. What I hear is “please give me amoxicillin”*,* “give me doxycycline”*,* “give me paracetamol.” And they do not even take a full dose. Instead*,* they take a half dose which is harmful because it only helps for some time and they become sick again. Later*,* when they go back and take a half dose again*,* they do not recover then they go to the hospital. Even if they are given a full dose they might not recover because they have been using it wrongly.” Human health care provider*,* Ngorongoro district FGD 07.*

Doctors pointed out that the practice of using medicines for purposes for which they are not needed, pressuring doctors to prescribe certain medicines, or self-treatment may be driven by a lack of knowledge of different types of medicines and how they function:

*“I think the main problem with the community is the knowledge of what an antibiotic is. A lot of drugs that are termed as antibiotics are not antibiotics. It’s quite a problem. There is an issue of colour*,* for example. Community members will tell you “rangi mbili”. When they say “rangi mbili”*,* they usually mean something like ampicillin*,* … [double-coloured capsules]*,* but sometimes you might find tramadol*,* a painkiller*,* has two colours too*,* yellow and green. It’s quite a challenge to explain what antibiotics are. I don’t know how to go about it because you have to share knowledge*,* I mean to inform them about bacteria*,* viruses*,* that they are different*,* they are treated differently*,* it’s quite a challenge.” Medical doctor*,* Referral facility*,* IDI 04.*

The quote demonstrates complexity in providing biomedical explanations about what antibiotics are to lay persons. It also exposes challenges in the language used, especially since biomedical terms in English may not have matching words in Swahili (the national language) or patient’s local languages.

#### Embodied disposition of livestock health care workers

Livestock health care workers described their activities as fraught with challenges, ranging from limited staff to farmer “preference” to treat their livestock on their own.

*“Many people in this village treat their livestock themselves because they know their livestock and they know the diseases… and due to the shortage of livestock officers.” Animal health care provider*,* Ngorongoro district*,* FGD 05.*

Having limited veterinary staff is problematic because it made access to veterinary services difficult as staff are unavailable when farmers need them.

#### Embodied dispositions of community members on human and livestock health

In agreement with observations made by health service providers, community members acknowledged that self-treatment in human beings and livestock was a common practice.

*“Maybe I do not have a health insurance card and I only have 2*,*000Tshs (0.9 USD) and I know that if I go to the dispensary it will cost me 6*,*000Tshs (2.6 USD). I will just go with my 2*,*000Tshs (0.9 USD) [to the pharmacy] and buy septrin.” Community member*,* human health FGD*,* Mwanga district*,* FGD 03.*

Table 2 provides additional reasons given for self-treatment. Antibiotics reportedly used for self-treatment were predominantly sourced from pharmacies, but some were remnant medicines from hospital treatment.

**Table 2:** Reasons provided by community members for self-treatment

Extrinsic reasons (outside the control of patients) • Unavailability of medicines in public health facilities • Lack of diagnostic services in some public health facilities • Lengthy distances to health facilities • Time consuming processes when seeking care in public health facilities occasioned by limited staff • Ineffective health insurance • Having easy access to pharmacies where medicines are available	Intrinsic reasons (within the control of patients) • To cut costs on healthcare • Previous successful experiences with self-treatment • Loss of trust in biomedical treatment after several rounds of treatment without recovery • Belief in local myths about biomedical treatment being harmful • Shame in disclosing what one is suffering from to medical staff • Desire to practice medical pluralism* (*utilizing more than one type of treatment simultaneously) • As “first aid” as one prepares to seek treatment in a health facility
**Medicines used for self-treatment**	
**Allopathic medicines**: • Antibiotics e.g. amoxicillin, doxycycline, pen V and flagyl • Antimalarials e.g. metakelfin, Artemether Lumefantrine (ALU) • Painkillers e.g. panadol, aspirin, paracetamol, hedex, diclophenac • Antihistamines • Cold relievers e.g. cofta, piriton, vicks	**Herbal medicines**: • Honey • Ginger • Garlic • Lemon • Aloe vera • *Myrisine africana* • *Warbugia ugandesis* • *Azandiratcha indica*

Community members reported self-treating their livestock (cattle, sheep, goats, pigs and poultry) regularly and livestock were treated with various veterinary products:

*“The most commonly available veterinary drugs purchased for private use by community members are pen-strep*,* tetracycline (oxytetracycline)*,* albendazole and ivermectin.” Animal health care providers*,* Mwanga district*,* IDI 01.*

Community members shared veterinary advice amongst themselves and sought new advice from veterinary shops. Use of antibiotics intended for human consumption such as amoxicillin capsules in poultry and pig disease management was also reported. Our findings on practices around antibiotic use in livestock at community levels are consistent with Caudell et al.’s (2017) study on antimicrobial use and veterinary care in northern Tanzania.

Animals that did not recover despite attempts to treat them were either sold or slaughtered for consumption. Participants did not report observing withdrawal periods before consumption of animal products from livestock undergoing treatment (consistent with Caudell et al. 2017). A medical doctor observed that there was a possibility of antibiotic overuse in broiler chicken production, exposing people to the risk of passive consumption of veterinary medicines. Some veterinary medicines are formulated from ingredients also used for treating human diseases, potentially contributing to ABR in humans.

*“Exotic chickens*,* most of us forget one thing*,* that they are given antibiotics for growth until they reach maturity. Most are broilers and for most the medicines that we use here [in the health facility] are the ones which they give them [chicken].” Medical doctor*,* Referral facility*,* IDI 03.*

Although farmers may be assumed to prefer to self-treat their animals as opposed to seeking professional services from veterinarians, it is worth noting that this “preference” is one borne out of necessity. In many LMICs, professional veterinary services are limited in terms of staffing and allied resources (Gauthier et al. 1999; Caudell et al. 2020; Davis et al. 2021). Pre- and post-colonially, farmers have utilized traditional/indigenous knowledges to manage their livestock’s health (Scoones and Wolmer 2006; Davis and Sharp 2020). Currently, these forms of disease management co-exist with biomedical forms of treatment and farmers may utilize either or both based on the knowledges they have acquired from experience (Kamat 2008; Langwick 2011; Davis and Sharp 2020).

As detailed above, the institutional and public discourse around AMR in Tanzania resonates with the global narratives which ascribe a moral value to the (mis)use of antibiotics. However, in revealing the social worlds within which health care providers, patients, pharmacists, and livestock farmers operate within– and the barriers to optimal health care provisions - a more complex picture emerges which pushes us to consider the “ethical moments” or “moral breakdown” behind antibiotic prescribing and use in Tanzania.

## Discussion

This paper reflects on the question of how morality is conveyed in the AMR discourse in Tanzania using Zigon’s (2010) framework of morality. Within our analysis of national and international policies around AMR and in the everyday practices and experiences of health practitioners, patients, and farmers we expose numerous aspects of morality and demonstrate how they are embedded in discourse, practice, and bodies. The 2017–2022 Tanzanian NAP exemplifies institutional policy discourse, framing AMR as a problem of undesirable behaviour in manufacture, distribution, dispensing, and prescribing. This discourse frames use of antimicrobials on a “lack”, be that a lack of knowledge, awareness, capacity, and so forth. This “lack” also situates AMR within an individualising moral discourse. To curb resistance, the NAP recommends awareness creation and behaviour change among actors creating, distributing, or using antimicrobials. Likewise, the public discourses and embodied dispositions held by health providers and community members frame AMR as a problem of behaviour, but alongside shortcomings in human and animal health infrastructure and service delivery.

The partial convergence in AMR discourse between institutional and human and animal health care providers demonstrates how different dimensions of morality are in dialogue with each other (Zigon 2010). The divergences between human and livestock health service providers and community members reveal complexity in institutional and public discourse revealing individual/place-based circumstances. These variations demonstrate the difficulties and potentially problematic outcomes when global institutions (e.g. WHO) or national governments imbue their discourse with moral talk/language. This can be mistakenly seen and accepted as a moral universalism, and one linked to important, life-saving acts. Exploring the broad anthropological (and sociological and philosophical) literature on moral universalisms is outside the scope of this paper (but see Beldo (2014) or Shweder (2004, 2012) or Fassin’s edited volume from 2012). Zigon’s approach to moral universalisms is one of caution, an approach we consider reasonable. Zigon also warns about seeing morality as either uniform *or* as solely relativistic. Morality does not just consist of “individual moral acts” (Beldo 2014: 272) in specific social contexts (e.g. prescribing antibiotics for care) but rather it is found throughout various aspects of the social world *and* it is articulated in discourse *and* is bound to local assemblages *and* is found in public, personal, and broader spheres (e.g. prescribing antibiotics without good diagnostics when a life is on the line, communicating dosing to patients in a language they may not fully understand, providing partial doses so a poor patient can get *some* help). Thus, Zigon’s morality is part of a system neither fully universal nor fully relativist but can provide contextual space (of either situational or universal spheres) (Scheweder 2012).

Thus, in invoking Zigon and in exploring the morality of AMR in our study setting, we reveal the “ethical moments” and moral dilemmas faced by a range of actors. It causes us to push back against the institutional discourse and potential universalising language of morality of AMR control. It also encourages us to ask critical questions about the morality, both hidden and in plain sight, within AMR discourse and practice, and how moral judgements come to be codified in policy and practice. Such questions have real world implications for practitioners and patients in their everyday decisions and on their bodies and health. For example, in LMICs, where testing capacity is limited, non-existent or unaffordable for patients (Horton et al. 2018; Sayed et al. 2018;), is a doctor wrong for ‘over’ prescribing antibiotics without confirmatory diagnosis? Where health facilities are distant and medical supplies there are inconsistent and costs deleterious (Chalker et al. 2015; Horumpende et al. 2018a, b), is a patient ‘wrong’ for practising self-treatment? When pharmacists sell partial antibiotic doses to patients, who is at “fault” - them for selling to earn their livelihood or the buyer for only buying what they can afford? Are farmers wrong in self-treating their animals in the absence of affordable and readily available professional veterinary services? Are attendants in veterinary shops mistaken in guiding farmers on how to treat their animals in the absence of professional veterinarians?

As decisions are made, there is a “disconnect between one’s moral ideals and real life ethical dilemma [which] forces one to step out of their everyday mode of moral being” (Zigon 2007: 144). While the actions taken by various actors may seem “irrational”, “imprudent”, or “sub-optimal” from a policy perspective, they may be quite the reverse in practice, particularly where medical alternatives are non-existent, and resources limited. What seems straightforward from a policy perspective might not be practical, particularly in under-resourced settings, and might serve to worsen the health outcomes of those with limited options. There needs to be a balance between protecting the vulnerability of human health and that of medicines threatened with resistance (Chandler et al. 2016). As Shweder’s careful exploration of morality in anthropology might suggest, these actions, acts, and choices may translate into a hierarchy of morality or moral acts. What gets condemned through policy should not ignore the everyday mode of actions taken by health providers. What is needed is not more regulations on antimicrobials but better response of the human and animal health systems to meet patient and provider needs.

The call for behaviour change among human and animal health actors may seem an obvious solution to reduce the risk of antibiotic resistance. It assumes that the current “imprudent”/ “sub-optimal”/ “irrational” practices are known and well understood, and actors can be urged to adopt “prudent”/ “optimal”/ “rational” practices. It further assumes that actors exercise absolute freedom of choice that is not inhibited or limited by intrinsic and extrinsic contextual factors in the prescription, dispensing, and use of medicines. This fails to address the fundamental question– what are the underlying factors that cause actors to act the way they do? Again, harkening back to Shweder’s (2004, 2012) scenarios of moral judgements and their justifications, are connections to context, chain of events, reasoning, agency, and “avoidance of physical or psychological harm” (2012: 98). In this study, patient pressure for antibiotics/medicine points to community perceptions of what constitutes medical care. It is not necessarily an “irrational behaviour”. The assumption that receiving medicine in a generic sense (whether antimicrobial or not) equals receiving care demonstrates the symbolic value medicines carry to patients. Chandler et al. (2016) point out that in their symbolic value, medicines can be used to validate one’s sickness; justify a patient’s adoption of the “sick role” and the care that comes with it; and empower the patient to exercise their sense of urgency over disease with the weapons they have in the form of medicines. Behaviours and practices can be indicators of underlying structural or systemic challenges which push people to act a certain way. Unless addressed, the call for behaviour change may remain futile. When asking people to change their behaviour, it is critical to have them know and understand why they need to change but most importantly to equip them with the means necessary to adopt desired changes. For example, Human Immunodeficiency Virus (HIV) studies demonstrate that increasing knowledge alone does not result in behaviour change (Durojaiye 2011; Oster 2012; Kaufman et al. 2014). If the global health community or national health providers are asking people to change behaviours, should we consider why we are asking this, and what the unintended consequences may be? This is particularly critical when these acts are moralised.

When health service providers articulated how their own actions as well as those of others (providers/patients) can be linked to AMR, they saw potential outcomes of individual actions. But while they asserted that some actions are undesirable and avoidable, others are a means of enhancing human and animal health within their resource-scarce settings. While discussions were tinged with a moralistic stance of “right” or “wrong” behaviours, there is also a recognition of the constraints that exist within the health system and the morality of acting in any capacity to improve health outcomes. What we see in the everyday health decisions that providers and patients make is that these are part of everyday actions that are “done” (Mahmood 2011) and by individuals with some sense of agency (Farnell 2000). Thus, actions (within a system of constraints) may often be un-reflexive of the conscious consideration of morality, and offer “different modalities through which the body comes to inhabit or live the regulative power of norms”, and more than dualistic aspects of health and morality (and beyond just resistance vs. constraints) (Mahmood 2011: 27). Specific kinds of bodily practice (like seeking health care or using antimicrobials) can draw lines between virtue and practice, and delineate the ethical subject (ibid.). The patients and farmers within our study do not necessarily view their own actions related to drug use from the moral, “right” vs. “wrong” perspective, but more often see drugs as a means to enhance their lives and that of their livestock, which is simply part of the everyday embodiment of health. Thus, they are making decisions and taking action while considering their own moral obligations to their family’s health. More investigation is needed in terms of assessing short versus long-term impacts of current antimicrobial use, however care can be taken now to address key issues exposed above.

The study poses the question whether morality has a role to play in antibiotic prescription, dispensing, and use in humans and livestock in East Africa. We found within the *institutional discourse* of AMR as seen in policy, language, and implementation AMR is framed as a moral problem of “right” or “wrong” behaviours to be corrected through individual behaviour change or punitive policies. *Public discourse* varies between patient, provider, and farmer but is also often imbibed with the language and sentiments of individual morality and “right” or “wrong” actions and choices. Finally, through prescribing practices, drug use, self-treatment, and care seeking, the *embodied dispositions* of AMR can affect health, bodies, beliefs, and health seeking behaviours. Embodied dispositions are not driven by thinking of “right” or “wrong” but what is perceived to bring greatest utility. This study concludes that what seems “imprudent”/ “irrational” from a policy perspective may be life enhancing in under-resourced settings. The call for behaviour change should not be used as a scapegoat to mask human and animal health system shortcomings and assign blame on prescribers, dispensers, and users. Thus, in addition to adopting policies on curbing antimicrobial resistance, supportive structures, services, and access for proper antibiotic use should be put in place to enable various actors to prescribe, dispense, and use medicine as needed.

### Strengths/limitations

The strength of this paper is in its novel approach to exploring AMR drawing into question the norms of policy, medical, and health intervention discourse. Our reflections on institutional and public discourses and embodied dispositions in every day antimicrobial use can challenge the sometimes problematic ways AMR gets discussed and addressed in public and policy spheres. These are useful in generating understanding of how low resource contexts experience and shape antimicrobials use and policy. On positionality, this study’s data collection was conducted by Tanzanian social scientists supervised by senior scientists from Tanzania, and non-Tanzanian social scientists with a long human and animal health research experience in the country, all of whom make the authorship of this paper. To eliminate bias in data analysis and interpretation, the team followed an iterative research process that included frequent reflection meetings to discuss emerging findings and subjected the findings to the broader project team for critique and AMR stakeholders at national and regional levels for validation. This study is not without limitations. The first is that although it was a study on antimicrobials, most of the findings relate to antibiotics. The second limitation is in not having pharmacists as key informants in this phase of the study to present their own views on antimicrobial use although we did include pharmacy attendants. Despite these shortcomings, the manuscript does provide insights into the role morality plays in antimicrobial use in humans and livestock.

## Electronic supplementary material

Below is the link to the electronic supplementary material.

Supplementary Material 1

Supplementary Material 2

Supplementary Material 3

Supplementary Material 4

Supplementary Material 5

## Data Availability

Data from which this paper has been developed is stored at the University of Glasgow’s data repository and is accessible upon request to social science lead Alicia.Davis@glasgow.ac.uk.
