# Supplementary material for: Antibiotic prescription, dispensing and use in humans and livestock in East Africa: does morality have a role to play?
Source: Monash Bioeth Rev. 2024 Oct 17;42(Suppl 1):125–49. doi: 10.1007/s40592-024-00208-z (PMC11850405; doi:10.1007/s40592-024-00208-z)
Supplement: Supplementary file 1 — Supplementary Material 1 [file 40592_2024_208_MOESM1_ESM.docx]

**Focus Group Discussion: Animal Health Care Providers**

1. In your community, where do people most often go for veterinary health care? (Follow up: Do they always go to professionals?) ***Ni wapi watu huenda mara nyingi kupata huduma ya afya za mifugo katika jamii hii? (Dadisi: Je huwa wanakwenda kwa wataalamu?)***
   1. How prevalent is ‘self-treatment’ of one’s own animals here? ***Ni kwa kiasi gani watu hupendelea kutibu mifugo wao wenyewe?***
   2. What do you as animal health professionals think about this? ***Wewe kama mtaalamu wa mifugo unafikiria nini juu ya hili (ya watu kutibu mifugo wao wenyewe)?***
2. What are the most common livestock health conditions/diseases that you see here in your community? (Enumerators: please have the respondents list ALL the veterinary health conditions that they see, and write them up on flip-chart paper) ***Katika jami yenu ni maradhi/magonjwa gani ya mifugo hutokea sana?***
3. Please have them rank from 1- to -5 the human conditions/illnesses that “concern” or “worry” them the most (1 is of most concern/worry). ***Tafadhali waelekeze kupangilia kati ya 1 mpaka 5 maradhi ya Mifugo ambayo yanawapa wasiwasi au hofu. (1 inayowapa wasiwasi/hofu zaidi)***
4. Go through each RANKED condition and ask:
   1. What is it about each ranked condition/illness that concerns them? Why? ***Ni ipi kuhusu/kwa nini _________ inakupa hofu?***
   2. What kind of diagnostics is available, and where (i.e. village, ward, district, region/zone; can be specific with EXACT duka/clinic name if available) ***Kuna aina gani ya uchunguzi/au vipimo inapatikana hapa, na wapi (kama ni: Kijiji, Kata, Wilaya, Mkoa/Kanda. Wanaweza kutaja jina la duka/kliniki maalumu kama inapatikana).***
   3. What kind of treatments are available? (if drugs, name the drugs) ***Ni aina gani ya matibabu yanapatikana? (kama ni dawa, taja hizo dawa)***?
   4. And where are each of these treatments available (i.e. village, ward, district, region/zone). ***Na ni wapi yanapatikana (kama ni Kijiji, Kata, Wilaya, Mkoa/Kanda)?***
   5. How much does the treatment cost (get a range of prices, exact number not critical) ***Matibabu hugharimu kiasi gani? -chukua bei mbalimbali, gharama halisi siyo lazima***
   6. Where do **farmers** go to get this treatment? ***Wafugaji/wakulima wanapendelea kwenda wapi kupata haya matibabu?***

- 1. Where do **you go as professionals** to get this treatment? What is the source of this treatment? ***Nyinyi kama wataalamu ni wapi mnaenda kupata matibabu haya? Nini chanzo cha haya matibabu***

Enumerator: fill in shorthand notes AFTER the interview ON THE INTERVIEW **FORM** with the information as follows. You can also create a small table on flip chart during

the interview to write a list of all the conditions, and then a separate page for the rankings. Once ranked 1-5 (1 as the most critical), you can go through a few key areas on flip chart to make sure the participants agree. Include Rank, Condition, Diagnostic, Treatment, Price, Locations

| Rank/Mpangilio | Condition/Hali | Why of concern? *Kwanini huleta wasiwasi?* | Type of Diagnostic (where is it available)? *Aina gani ya uchunguzi (unapatikana wapa?)* | Treatment (drugs or other things besides drugs) | Price Range/Bei | Where do patients get this treatment?/Wagonjwa wanapata wapa hizi dawa? | Where do you as professionals get this treatment? Ni wapi wewe kama mtaalamu unapata haya matibabu? |
| --- | --- | --- | --- | --- | --- | --- | --- |
| 1. |  |  |  |  |  |  |  |

1. What are the most commonly available veterinary drugs for **private** purchase (from an agro-vet shop, market or shop) in this community? ***Ni dawa gani za mifugo za kawaida zinapatikana kwa wanunuzi binafsi (kutokana na duka la dawa za mifugo, soko, au duka) katika jamii hii?***
2. What are the most commonly available veterinary drugs for administering by LFOs/government veterinarians in this community? ***Ni dawa gani za mifugo zinazopatikana kawaida ambazo hutolewa na daktari ya mifugo ya serekali (LFO).***
   1. **Where do you get these drugs from? *Dawa hizi mnapata kutoka wapi?***

*[enumerators may want to list the drugs on a flip chart and include ‘where’ next to the drug name]*

**HEALTH CAMPAIGNS:** We now want to ask you some questions about health campaigns in your community: ***Sasa tutawauliza maswali kuhusu kampeni za afya katika jamii yenu:***

1. What makes a good/effective health campaign? And Why? ***Ni kitu gani kinafanya kampeni ya afya kuwa nzuri/ na yenye ufanisi? Na kwanini?***
2. What makes a bad health campaign and why? ***Ni kitu gani kinasababisha kampeni ya afya kuwa mbaya na kwanini?***
3. Have there been health campaigns here in this village in the past? (What was the health issue? what do people remember about it? what messages stuck and why?). ***Katika kipindi kilichopita kumewahi kuwa na kampeni ya afya katika kijiji hiki? (ilikuwa inahusiana na suala gani la afya? Watu wanakumbuka nini kuhusu hilo? ujumbe gani umeshikilia zaidi? Kwanini?***
4. When was the last health campaign? Ni lini mara ya mwisho kuwa na ***kampeni ya afya?***
5. Was it part of a national campaign? ***Ilikuwa ni sehemu ya kampeni ya taifa?***
6. What information was disseminated and how? ***Ni taarifa zipi zilisambazwa? Ilizambazwa kwa namna gani?***
7. What was the key message delivered by the campaign? ***Ujumbe gani mkuu ulitolewa na kampeni hiyo?***
8. Do you think this was an effective campaign? ***Why/Why not? Unafikiri ilikuwa kampeni yenye ufanisi? Na kwanini?***
