# Supplementary material for: Antibiotic prescription, dispensing and use in humans and livestock in East Africa: does morality have a role to play?
Source: Monash Bioeth Rev. 2024 Oct 17;42(Suppl 1):125–49. doi: 10.1007/s40592-024-00208-z (PMC11850405; doi:10.1007/s40592-024-00208-z)
Supplement: Supplementary file 2 — Supplementary Material 2 [file 40592_2024_208_MOESM2_ESM.docx]

**Focus Group Discussion—Community Members**

**TYPE FGD: HUMAN HEALTH**

**QUESTIONS**

1. How do you define “health”? ***Nini maana ya neno “Afya”? (unaelewa nini kuhusu afya au tukisema neno “afya”, unaelewa nini)?***
   1. Is there a difference between “health” and “good health”? ***Kuna tofauti gani kati ya afya na afya nzuri?***
2. What are the top 5 characteristics that help you determine whether or not a person is “healthy”? ***Ni sifa zipi kuu 5 ambazo zinaweza kukusaidia kutambua iwapo mtu ana afya au hapana.***
   1. What are the top 5 characteristics that help you determine whether or not a person is “unhealthy/ill”? ***Na ni zipi tabia tano zinazo kusaidia kutambua/kujua kama watu hawako wazuri/wana hali mbaya?***
3. How do you define *wellbeing “good life”* maisha mazuri*? (mzima/uzima)* ***Nini maana ya “maisha mazuri”? (unaelewa nini tukisema “maisha mazuri”)?***
4. Where do you normally get information about health issues (for yourself, family, community)? **Ni wapi kikawaida unapata habari kuhusu huduma za kiafya? (kwa wewe, familiya/kaya, na jamii).** [Enumerators: try and collect at least 3-5 sources] When/where/how/what kind of information? **wakati/ wapi/ kivipi/ aina gani ya habari?**
5. **Of these mentioned,** whom do you most trust as **sources of information about human health? Kwa hizo zilizotajwa, ni nani au zipi unaziaamini kuwa chanzo cha taarifa kuhusu afya?**
   1. Why? What is it about the information/people (what you just mentioned) that leads you to trust it/them? **Ni nini kimekupelekea wewe kuwaamini hao?**
6. In your community, who do you go to for specific advice for your health problems? Why? **Katika jamii, ni nani unaenda kumuona kwa ajili ya ushauri maalum kuhusu matatizo ya afya wako? Kwanini?**
7. What resources do people in this community have for health care? (please make sure to note if inside or outside the village/ward/district and where specifically) **Ni rasilimali au huduma za afya gani zinazopatikana katika jamii yenu? (ndani au nje ya kijiji/kata/wilaya na ni wapi?)**
   1. Of those named, which are the ones you most often use? **Kati ya hizo, zilizotajwa ni huduma zipi zinatumika mara kwa mara/zaidi?**
   2. (Follow up only if NOT MENTIONED: Do you usually go to professionals?) **(Je, unakwenda kwa wataalam?)**
   3. (Do you go within or outside the village/ward/district?) **Je, unaenda nje au ndani ya kijiji/kata/wilaya kufuata huduma?**
   4. What kind of health care providers, what specific clinics/hospitals? **Ni aina gani ya huduma wanatoa, ni hospitali gani mahususi/ hospitali? (ni ya serekali, ya binafsi, ya kanisa, ni zahanti, kliniki, hospitali?)**
   5. Can you name them specifically **Unaweza kututajia majina za huduma au watu wa huduma zinazopatikana hapa?**
8. How prevalent is ‘self-treatment’? ) ***Ni kwa kiasi gani watu hupendelea kujitibu mwenyewe?***
9. What kinds of medicines do people commonly use and where do people get them? **Ni dawa gani watu wanatumia sana sana na ni wapi watu wanapata hizo?**
10. What are the most common human health conditions/illnesses in your community? (Enumerators: please have the respondents list ALL the health conditions that they see, and write them up on flip-chart paper)

***Katika jamii yenu ni maradhi/magonjwa gani hujitokeza sana katika afya ya binadamu? (muongozaji: Wape nafasi washiriki kutaja hali za kiafya wanazoziona, orodhesha kwenye karatasi (flip chat)***)

1. Please have them rank from 1- to -5 the human conditions/illnesses that “concern” or “worry” them the most (1 is of most concern/worry). ***Tafadhali waelekeze kupangilia kati ya 1 mpaka 5 maradhi ya binadamu ambayo yanawapa wasiwasi au hofu (1 inayowapa wasiwasi/hofu zaidi).***
2. Go through each RANKED condition and ask: ***Pitia kila hali iliyopangiliwa na uliza:***
   1. What is it about ___________~~(~~ranked conditions/illness) that concerns them? Why? ***Ni ipi kuhusu/kwa nini _________ inakupa hofu?*** *(Don’t need to write this down).*
   2. What do you do when you think you have this condition? ***Utafanya nini kwa wakati huo ukifikiri una hali hiyo?*** *(Don’t need to write this down).*
   3. What options do you have for treatment? What kinds of treatments are available? (if drugs, name the drugs; if health facility, name health facility) ***Ni aina gani ya matibabu yanapatikana? (kama ni dawa, taja hizo dawa; kama aina nyingine ya matibabu taja)*.**
   4. Where do **you** go to get this treatment? ***Ni wapi unaenda kupata matibabu?***
   5. How much does the treatment cost (get a range of prices, exact number not critical) ***Matibabu yanagharimu kiasi gani? (pata bei mbali mbali halisi siyo lazima).***
