# Supplementary material for: Antibiotic prescription, dispensing and use in humans and livestock in East Africa: does morality have a role to play?
Source: Monash Bioeth Rev. 2024 Oct 17;42(Suppl 1):125–49. doi: 10.1007/s40592-024-00208-z (PMC11850405; doi:10.1007/s40592-024-00208-z)
Supplement: Supplementary file 3 — Supplementary Material 3 [file 40592_2024_208_MOESM3_ESM.docx]

**Focus Group Discussion Human Health Care Providers**

**QUESTIONS:**

1. Where do people most often go for health care in your community? (Follow up: Do they always go to professionals/clinics? ***Ni wapi watu huenda mara nyingi kupata huduma ya afya katika jamii hii? (Dadisi: Je huwa wanakwenda kwa wataalamu/matabibu?)***
   1. How prevalent/how often do people ‘self-treat’ here? ***Ni kwa kiasi gani watu hupendelea kujitibu mwenyewe?***
   2. What do you as health professionals think about this? ***Wewe kama mtaalamu wa afya unafikiria nini juu ya hili (ya watu kujitibu mwenyewe)?***
2. What are the most common human health conditions/illnesses that you see here in your community? (Enumerators: please have the respondents list ALL the health conditions that they see, and write them up on flip-chart paper)

***Katika jamii yenu ni maradhi/magonjwa gani hujitokeza sana katika afya ya binadamu? (muongozaji: Wape nafasi washiriki kutaja hali za kiafya wanazoziona, orodhesha kwenye karatasi (flip chat)***)

1. Please have them rank from 1- to -5 the human conditions/illnesses that “concern” or “worry” them the most (1 is of most concern/worry). ***Tafadhali waelekeze kupangilia kati ya 1 mpaka 5 maradhi ya binadamu ambayo yanawapa wasiwasi au hofu. (1 inayowapa wasiwasi/hofu zaidi)***
2. Go through each RANKED condition and ask: ***Pitia kila hali iliyopangiliwa na uliza:***
   1. What is it about ___________~~(~~ranked conditions/illness) that concerns them? Why? ***Ni ipi kuhusu/kwa nini _________ inakupa hofu?***
   2. What kind of diagnostics is available, and where (i.e. village, ward, district, region/zone; can be specific with EXACT duka/clinic name if available) ***Matibabu yakoje? Kuna aina gani ya uchunguzi/au vipimo zinazopatikana hapa, wapi (kama ni: Kijiji, Kata, Wilaya, Mkoa/Kanda. Wanaweza kutaja jina la duka/kliniki maalumu kama inapatikana). NEED PLURAL***
   3. What kind of treatments are available? (if drugs, name the drugs) ***Ni aina gani ya matibabu yanapatikana? (kama ni dawa, taja hizo dawa)***
   4. And where all the possible places its available to people in this village (i.e. village, ward, district, region/zone)? ***Na ni wapi (kama ni Kijiji, Kata, Wilaya, Mkoa/Kanda)***
   5. How much do the diagnostics and treatments cost (get a range of prices, exact number not critical, but make sure to capture the cost of both the diagnostics and the treatments). ***Uchunguzi na matibabu hugharimu kiasi gani? ( chukua bei mbalimbali, gharama halisi siyo lazima)***
   6. Where do **patients** most often go/prefer to get this treatment? ***Wagonjwa wanapendelea kwenda wapi kupata haya matibabu?***
   7. Where do **you go as professionals** to get/procure this treatment? What is the source of this treatment? ***Nyinyi kama wataalamu ni wapi mnaenda kupata matibabu haya? Nini chanzo cha haya matibabu?***

Enumerator: fill in shorthand notes AFTER the interview with the information as follows. You can also create a small table on flip chart during the interview to write a list of all the conditions, and then a separate page for the rankings. Once ranked 1-5 (1 as the most critical), you can go through a few key areas on flip chart to make sure the participants agree. Include Rank, Condition, Diagnostic, Treatment, Price, Locations

| Rank/mpangili | Condition/hali | Why of concern?/ *Kwanini huleta wasiwasi?* | Type of Diagnostic (where is it available)? *Aina ya uchunguzi (unapatikana wapi?)* | Treatment  (drugs or other things besides drugs)/matibabu | Where are all the places this treatment is Available for patients? | Price Range (specify if drugs or not drugs)/bei | Where do patients prefer to go to get this treatment?/ *Ni wapi wagonjwa wanapata matibabu?* | Where do you as professionals get this treatment?/ Ni wapi wewe kama mtaalamu unapata haya matibabu? |
| --- | --- | --- | --- | --- | --- | --- | --- | --- |
| 1. |  |  |  |  |  |  |  |  |

1. What are the most commonly available human drugs for **private** purchase *from a regular shop or pharmacy* in this community? ***Ni dawa gani za binadamu za kawaida zinapatikana kwa wanunuzi binafsi kutoka duka la kawaida au duka la dawa katika jamii hii?*** Na wapi? (names of the dukas, how many in the community, is it in the community).
   1. Do people really need an Rx to get this drug in your community? ***Je, ili kupata hizi dawa ni lazima kuwa na karatasi iliyoandikwa na daktari?***
2. What are the most commonly prescribed drugs by health providers (in this community? ***Ni dawa gani za binadamu unawandikia wagonjwa mara kwa mara katika jamii hii.***
   1. Do people really need an Rx to get this drug in your community? ***Je, hawa wagonjwa wanahitaji kupata karatasi iliyoandikwa na daktari kwenda kununua hizo dawa?***
   2. **Where do you get these drugs from?** *[enumerators may want to list the drugs on a flip chart and include ‘where’ next to the drug name]* ***Dawa hizi mnapata kutoka wapi?***

**HEALTH CAMPAIGNS:** We now want to ask you some questions about health campaigns in your community: ***Sasa tutawauliza maswali kuhusu kampeni za afya katika jamii yenu:***

1. What makes a good/effective health campaign? And Why? ***Ni kitu gani kinafanya kampeni ya afya kuwa nzuri/ na yenye ufanisi? Na kwanini?***
2. What makes a bad health campaign and why? ***Ni kitu gani kinasababisha kampeni ya afya kuwa mbaya na kwanini?***
3. Have there been health campaigns here in this village in the past? (what was the health issue, what do people remember about it, what messages stuck and why) ***Katika kipindi kilichopita kumewahi kuwa na kampeni ya afya katika kijiji hiki? (ilikuwa inahusiana na suala gani la afya? Watu wanakumbuka nini kuhusu hilo? ujumbe gani umeshikilia zaidi? Kwanini?***
   1. When was the last health campaign? ***Ni lini mara ya mwisho kuwa na kampeni ya afya?***
   2. Was it part of a national campaign? ***Ilikuwa ni sehemu ya kampeni ya taifa?***
   3. What information was disseminated and how? ***Ni taarifa zipi zilisambazwa? Ilizambazwa kwa namna gani?***
   4. What was the key message delivered by the campaign? ***Ujumbe gani mkuu ulitolewa na kampeni hiyo?***
   5. Do you think this was an effective campaign? ***Why/Why not? Unafikiri ilikuwa kampeni yenye ufanisi? Na kwanini?***
