# Supplementary material for: Antibiotic prescription, dispensing and use in humans and livestock in East Africa: does morality have a role to play?
Source: Monash Bioeth Rev. 2024 Oct 17;42(Suppl 1):125–49. doi: 10.1007/s40592-024-00208-z (PMC11850405; doi:10.1007/s40592-024-00208-z)
Supplement: Supplementary file 4 — Supplementary Material 4 [file 40592_2024_208_MOESM4_ESM.docx]

**Focus Group Discussion—Community Members**

**TYPE FGD: ANIMAL HEALTH**

1. How do you define “health”? ***Nini maana ya neno “Afya”? (tukisema neno “afya”, unaelewa nini)?***
   1. Is there a difference between “health” and “good health”? **Kuna tofauti gani kati ya afya na afya nzuri?**
   2. What are the top 5 characteristics that help you determine whether an animal is “healthy”? ***Ni sifa zipi kuu 5 ambazo zinaweza kukusaidia kutambua iwapo mnyama ana afya nzuri?***
   3. What are the top 5 characteristics that help you determine whether an animal is “unhealthy/ill”? ***Ni sifa zipi kuu tano ambazo zinaweza kukusaidia kutambua iwapo mnyama wako hana afya nzuri?***
2. Where do you normally get information about livestock health issues (at community and individual levels)*?* ***Ni wapi kikawaida unapata habari kuhusu taarifa kuhusiana na afya ya mifugo (kwa jamii kwa ujumla na kwa kaya/boma)?***  [Enumerators: try and collect at least 3-5 sources] When/where/how/what kind? ***Wakati/ wapi/ kivipi/ aina gani ya habari?***
3. **Of these mentioned,** whom do you most trust as **sources of information about animal/livestock health**? ***Kwa hizo zilizotajwa, ni nani au zipi unaziaamini kuwa chanzo cha taarifa kuhusu afya ya mifugo?***
   1. Why? What is it about the information/people (what you just mentioned) that leads you to trust it/them? ***Ni nini kimekupelekea wewe kuwaamini hao?***
4. In your community, who do you go to for specific advice for your livestock’s health problems? Why? **Katika jamii, ni nani unaenda kumuona kwa ajili ya ushauri maalum kuhusu matatizo ya afya ya mifugo yenu? Kwanini?**
5. What resources do people in this community have for veterinary care or for livestock services? (please make sure to note if inside or outside the village/ward/district) **Ni rasilimali gani au huduma za afya gani kwa mifugo zinazopatikana katika jamii yenu? (? (ndani au nje ya kijiji/kata/wilaya na ni wapi?)**
   1. Are these **all/only** livestock resources available to you? If so, what other livestock resources are available? ***Kuna rasilimali zingine (za mifugo) katika hii jamii? Kama zipo tunaomba utuelezee ni zipi?(***
   2. Can you name them specifically ***Unaweza kututajia majina za huduma au watu wa huduma zinazopatikana hapa?***
   3. Of those named, which are the ones you most often use? ***Kati ya hizo, zilizotajwa ni huduma zipi zinatumika mara kwa mara/zaidi?***
   4. (Follow up **only if NOT MENTIONED**: Do you usually go to professionals?) ***(Je, unakwenda kwa wataalam?)***
   5. (Do you go within or outside the village/ward/district?) ***Je, unaenda nje au ndani ya kijiji/kata/wilaya kufuata huduma?***
   6. How prevalent is ‘self-treatment’ of livestock here? ) ***Ni kwa kiasi gani watu hupendelea kutibu mifugo wenyewe?***
6. **IF NOT ANSWERED ABOVE**: What specific veterinary infrastructure exists, what are they called? ***Kama hawajajibiwa 7 uliza: Kuna miundo gani mahususi kwa huduma za mifugo (kama: duka la dawa ya mifugo, LFO, sokoni, CAHWS)? Wanaitwaje?***
7. What are the most common livestock diseases (for your livestock) in your community? ***Ni magonjwa gani ya mifugo hutokea mara kwa mara katika hii jami?*** (Enumerators: please have the respondents list ALL the animal health conditions that they see, and write them up on flip-chart paper) .
8. Please have them rank from 1- to -5 the animal health conditions/diseases that “concern” or “worry” them the most (1 is of most concern/worry). ***Tafadhali waelekeze kupangilia kati ya 1 mpaka 5 maradhi ya mifugo ambayo yanawapa wasiwasi au hofu. (1 inayowapa wasiwasi/hofu zaidi).***
9. Go through each RANKED condition and ask: ***Pitia kila hali iliyopangiliwa na uliza:***
   1. What is it about ___________~~(~~ranked conditions/illness) that concerns them? Why? ***Ni ipi kuhusu/kwa nini _________ inakupa hofu?***
   2. What do you do when you think your animals have this condition? ***Unafanya nini unapokifiri mifugo yako ina hali hiyo?***
   3. What options do you have for treatment? What kind of treatments are available? (if drugs, name the drugs; if other than drugs, name the treatment) ***Ni aina gani ya matibabu yanapatikana? (kama ni dawa, taja hizo dawa; kama aina nyingine ya matibabu taja).***
   4. Where do **you** go to get this treatment? ***Ni wapi unaenda kupata matibabu?***
   5. How much does the treatment cost (get a range of prices, exact number not critical) ***Matibabu yanagharimu kiasi gani? (pata bei mbali mbali halisi siyo lazima).***
