# Supplementary material for: Antibiotic prescription, dispensing and use in humans and livestock in East Africa: does morality have a role to play?
Source: Monash Bioeth Rev. 2024 Oct 17;42(Suppl 1):125–49. doi: 10.1007/s40592-024-00208-z (PMC11850405; doi:10.1007/s40592-024-00208-z)
Supplement: Supplementary file 5 — Supplementary Material 5 [file 40592_2024_208_MOESM5_ESM.docx]

**“Supporting the National Action Plan for Antimicrobial Resistance” SNAP-AMR**

Project Number 303374

**Health Care Providers Interview Schedule**

N.B. This will be qualitative research and so specific questions may change. This interview schedule provides the questions that will be asked in all interviews, as well as indicative themes and example questions that will inform the qualitative interviews that will be semi-structured, and thus, may alter depending on the responses of the research participant.

# General Information

Date:_________________

Name of Researcher: ______________________________________

Name of Zone: ____________________________________________

Name of Health Facility: _____________________________________

Name of Facility Ward: ____________________________________________

Interview Research Participant Number: ________________________________

Position of Participant Giving Information:

1. Medical Doctor Specialist [Please specify]: __________
2. Medical Doctor Resident [Please specify]: __________
3. Medical Doctor Registrar:
4. Medical Intern [ ]
5. Clinical Officer [ ]
6. Assistant Medical Officer [ ]
7. Registered Nurse [ ]
8. Enrolled Nurse [ ]
9. Nurse Midwife [ ]
10. Nurse Attendance [ ]
11. Other (please specify) [ ] __________________

# theme: Personal motivations and EXPERIENCES working within the health system

Indicative questions include:

1. How long have your worked in the health system?
2. What motivated you to work in the health system in Tanzania?
3. What do you enjoy most about working in the health system?
4. What are the biggest challenges to working in the health system in Tanzania?
5. How do you think these can be overcome?
6. What do you consider the three most important health priorities in the Tanzania, presently?
7. In as much detail as possible, can you talk through what your average day is like in your role? (i.e. length/ when do you see patients/how many do you see)
8. How do you feel at the end of your shift?
9. What are your hopes and fears for the Tanzanian health system in the next decade?

# Theme: amr Knowledge and Attitude

Indicative questions include:

1. What do you understand as ‘Anti-microbial resistance’ to mean?
2. Do you consider AMR a problem in your health facility, and why?
3. Do you consider AMR a problem in Tanzania, and why?
4. Do you consider AMR a problem globally, and why?
5. What do you think the main drivers of AMR are in your health facility?
6. What do you think the main drivers of AMR are in Tanzania?
7. What do you think the main drivers of AMR at a global level?
8. As a health care practitioner, do you feel your individual behavior can influence AMR (either the spread of it or the fight against it)?
9. Do you think antibiotics are overused in your health facility, why?
10. Do you think antibiotics are overused in community, why?
11. What are your experiences of AMR within your health facility?
12. Is AMR something you worry about regularly?
13. How does it feel as a health professional to be facing a growing AMR problem?
14. What do you think your job without effective antibioitcs would be like, and do you think you will face these challenges?
15. How do you balance the needs of current patients you are treating with the need of future patients?
16. Are you aware of the Tanzanian National Action Plan on Antimicrobial Resistance?

If so,

- 1. What are your thoughts about it?
  2. Has it changed your current practice in any way, and if so how?

1. Who is or should be responsible for tackling AMR?

# theme: choice and prescribing practices

Indicative questions include:

1. What are the main things that influence your prescribing practices of antibiotics?
2. When seeing a patient, how do you decide if they need a prescription for antibiotics, and what antibiotics to prescribe? (i.e. do you discuss with colleagues/ run tests/ clinical history/official guidelines/drug availability/ request from patients/pressure for drug representatives)
3. What sort of antibiotics are you regularly prescribing?
4. What are the most common ailments you see that would make you prescribe antibiotics?
5. Drug availability - Does your facility always have all the suitable antibiotics or are there times when certain antibiotics are not available?
6. What do you do if they do not have your preferred antibiotics in stock?
7. How do you feel if stock is unavailable?
8. Are you able to take a full patient history before prescribing antibiotics or are there too many time constraints or challenges?
9. Can you see patient’s medical records to see their previous visits, and does this inform your prescribing?
10. Do patients put you under pressure to prescribe them antibiotics? If so, in what way, how do you respond and how does that make you feel?
11. How does the pressure or prescribing practice differ between insured and non-insured patients?
12. If you refuse to prescribe antibiotics, how does your patient respond? Do you think they go elsewhere to get antibiotics?
13. How do time pressures and high patient numbers influence how you interact/diagnose/prescribe drugs to your patient?
14. When would you prescribe broad spectrum antibiotics and when would you prescribe narrow spectrum?
15. What diagnostic tests are available to you? (can you regularly take blood tests/urine samples/ cultures/drug sensitivity testing?)
16. Do you regularly seek further tests before prescribing antibiotics, or is this not practical, and why?
17. How does the availability of diagnostic tests alter your prescribing behavior?
18. When would you undertake diagnostic testing? What factors would influence this decision [i.e. symptoms/ patient’s history/ age /availability of tests/patient’s ability to pay?]? Are these tests done on site or are they proeccessed elsewhere?
19. How long does it take to receive test results, and do you prescribe antibiotics while waiting for the results to come back?
20. What improvements to testing are needed before you systematically test before prescribing antibiotics [i.e. faster results/ cheaper tests/ improved availability of equipment/ more staff/ more time to spend with patient]?
21. What do you do if you have an uninsured patient who can’t afford to pay for diagnosis?
22. For inpatients, when do you stop antibiotic use (i.e. receiving test results/patient improves/patient’s condition gets worse)
23. Do you ever consider AMR when prescribing antibiotics?
24. Are counter fit drugs an issue in Tanzania?
25. Do patients ever report using animal drugs on themselves when ill?
26. Are there situations you would use antibiotics as prophylaxis rather than curatively? And if so, give example.

# theme: communication with patients

Indicative questions include:

1. Do you think your patients are aware of AMR?
2. Do you communicate about AMR with patients, and if so, how?
3. How do your patients respond to the messages being communicated?
4. What do you consider the biggest challenge in communicating AMR to patients?
5. What do you think can be done in order to improve communication with your patients?

# theme: Infection Prevention control

Indicative questions include:

1. What do you consider the main challenges to employing successful IPC practices in your health facility?
2. Have you received handwashing training? If so, have you implemented it into your practice? What are the challenges of implementing it into your practice?
3. Are the resources to employ successful IPC practices available in your health facility?
4. Do you feel you have the time to employ successful IPC practices in your health facility?

# theme: Information flows

Indicative questions include:

1. Who or what do you consult if you are seeking advice on prescribing antibiotics? Do you trust the information?
2. During a shift, do you have time to discuss with other colleagues around antibiotic use and does this alter your practice?
3. Do you know if your facility has an AMR steward, and if so, what is their role and what do you think of it?
4. Have you had the opportunity for AMR CPD, and if so, what did you think of it?
5. Would you like to receive more training or information on AM use and AMR? If so, why do you think it is important? What format would be most useful?
6. As a health care professional, what do you think the priorities need to be in order to tackle the AMR problem?
7. If you could change one thing with about the Tanzanian health system what

# theme: Procrumrment Practices (NOT FOR DOCTORS)

Indicative questions include:

1. Can you talk through how you procure drugs for the hospital?
2. What are your thoughts on this system?
3. What works within this system, and what are the major challenges?
4. Do you always receive all the medication you order, and if not, what do you do?
5. Do you receive donations of drugs? If so how frequently, from who and how much and how are they processed? What sort of medication do you get donated, are these donations a result of specific request?
6. How could the procurement process be improved in Tanzania?
7. How is the drug budget managed within the zone/facility?
8. Is equipment and goods for IPC (soaps, cleaning material) procured in the same way as drugs?
9. Are IPC goods procured privately or through government channels?
